# Cross-Sectional Associations of Cognitive Function, Frailty, Depressive Symptoms, and Self-Care Dependence in Residents of a Long-Term Care Institution

**DOI:** 10.3390/healthcare14152428

**Published:** 2026-08-06

**Authors:** Xiaorong Gao, Shiqi He, Wenli Zhang, Xiya Pan, Keke Chen, Qiaoqiao Wang

**Affiliations:** 1 School of Medicine, Hangzhou City University, Hangzhou 310015, China; 2250408006@stu.hzcu.edu.cn (X.G.); 32504314@stu.hzcu.edu.cn (X.P.); 32504298@stu.hzcu.edu.cn (K.C.); 2 Hangzhou First People’s Hospital, Hangzhou 310006, China; 32404300@stu.hzcu.edu.cn; 3 Hangzhou Third Social Welfare Institute, Hangzhou 310021, China; hzfly2011@126.com

**Keywords:** cognitive function, cognitive screening, physical frailty, depressive symptoms, activities of daily living, self-care dependence, cross-sectional regression decomposition, long-term care, older adults

## Abstract

**Highlights:**

**What are the main findings?**

- Among 182 residents of one long-term care institution, lower cognitive scores co-occurred with greater self-care dependence, higher FRAIL scores, and more GDS-15 symptoms. The prespecified regression decomposition describes associations only and does not identify a clinical sequence, mechanism, or intervention target.
- Reversing the order of frailty and depressive symptoms produced another compatible decomposition of the same cross-sectional covariance structure. Neither ordering establishes temporal precedence or a causal pathway.

**What are the implications of the main findings?**

- Frailty and depressive symptoms are clinically relevant correlates that warrant prospective study. The present analysis does not show that modifying either condition will preserve self-care.
- The association structure varied with the specified ordering and residential composition. Across six building-omission analyses, all three path-product quantities retained their direction but changed in magnitude. These descriptive ranges are not cluster-robust inference; longitudinal, multicenter studies are needed before temporal or care-sequence implications can be considered.

**Abstract:**

**Background:** Cognitive and functional difficulties commonly coexist in long-term care. Frailty and depressive symptoms may also co-occur with cognitive function and self-care dependence, but cross-sectional data cannot determine temporal order. We characterized the association structure among these measures. **Methods:** We analyzed complete standardized assessments from 182 residents of one long-term care institution. Cognitive function (Mini-Mental State Examination), physical frailty (FRAIL scale), depressive symptoms (15-item Geriatric Depression Scale), and self-care dependence (20-item Chinese Activities of Daily Living scale; higher scores indicate greater dependence) were assessed. Prespecified regression equations were adjusted for age, sex, and education. Path-product quantities and participant-resampling bias-corrected intervals (BC, not BCa) were used as descriptive stability summaries, not cluster-robust confidence intervals. Residential heterogeneity was examined with intraclass correlations and leave-one-building-out estimates. **Results:** Lower cognitive scores co-occurred with greater frailty, more depressive symptoms, and greater dependence. In the adjusted model, the total cognition–self-care association was c = −1.501 (BC interval −1.716 to −1.274), and the conditional association coefficient was c′ = −1.082 (−1.357 to −0.779). The frailty, depression, and ordered path-product quantities were −0.221, −0.166, and −0.033, respectively. Self-care dependence had a cohort intraclass correlation of 0.42. Across six building-omission analyses, the ordered quantity ranged from −0.050 to −0.018 and the frailty quantity from −0.272 to −0.164. **Conclusions:** These findings describe interrelated measures within one institution. They do not establish causal mediation, temporal order, or an intervention sequence.

## 1. Introduction

China’s aging population has increased reliance on institutional long-term care. Residents of these facilities are often older, medically complex, and cognitively vulnerable [1,2]. Reduced ability to perform activities of daily living is an important correlate of cognitive difficulty and is associated with more intensive care, higher costs, poorer quality of life, and mortality [3]. Identifying modifiable conditions that co-occur with cognitive and functional difficulty may therefore help define hypotheses for longitudinal and interventional research.

Lower cognitive function is consistently associated with greater dependence in daily activities [4,5], but it does not explain all between-person variation in functional ability [6]. Physical frailty and depressive symptoms are two plausible co-occurring conditions. Here, physical frailty denotes reduced physiological reserve and greater vulnerability to stressors, measured through fatigue, resistance, ambulation, illnesses, and weight loss on the FRAIL scale [7,8]. Psychological frailty was not included. Depressive symptoms may accompany disability through reduced motivation, psychomotor slowing, and limited participation in daily tasks [9]. The GDS-15 measures symptom burden and does not establish a depressive-disorder diagnosis.

Frailty and depressive symptoms have usually been examined as related but distinct correlates of cognition and disability [10,11]. A serial regression model can decompose observed associations under a specified ordering, but cross-sectional data cannot show that one intervening measure precedes another. Studies in community and nursing-home samples have used different parallel or serial specifications for these constructs [12,13,14,15]. Their results illustrate both the clinical relevance of the measures and the dependence of interpretation on model specification. Alternative orderings should therefore be treated as competing descriptions, not causal mechanisms.

A frailty-first ordering is clinically plausible but is not uniquely implied by the data. Cognitive and physical impairment overlap within the broader concept of cognitive frailty [16,17,18,19]. Frailty may accompany depressive symptoms, whereas low mood may reduce activity and contribute to frailty. Longitudinal studies have reported bidirectional associations [20,21], and a 2026 cross-national cohort study found that baseline frailty predicted incident depressive symptoms [22]. This evidence motivated the primary ordering but did not validate it. We therefore measured the four constructs separately and examined construct overlap and alternative ordering in sensitivity analyses.

We studied residents of one long-term care institution and specified cognitive function as the exposure measure, frailty and depressive symptoms as ordered intervening measures, and self-care dependence as the outcome measure. This ordering defined algebraic path-product quantities only. We also evaluated a depression-first ordering and building-omission sensitivity to assess dependence on model specification and residential composition.

## 2. Materials and Methods

### 2.1. Study Design and Setting

We conducted this cross-sectional study at the Hangzhou Third Social Welfare Institute, a public long-term care institution in Hangzhou, China, including its dedicated cognitive-impairment care unit.

This cross-sectional analysis used standardized questionnaire assessments and institutional records collected between December 2025 and March 2026. All 182 questionnaires were administered after the Medical Ethics Committee of Hangzhou City University approved the study protocol (HZCU2025-30; 11 December 2025). Each participant personally provided written informed consent specifically for the cross-sectional questionnaire study before assessment. At the time of data collection, no participant had been enrolled in or randomized to the separately planned intervention study, and no intervention had been delivered. The planned intervention study is currently undergoing public registration and is outside the scope of this report. Reporting followed the Strengthening the Reporting of Observational Studies in Epidemiology (STROBE) guideline for cross-sectional studies [23].

### 2.2. Participants

The analytic dataset comprised 182 permanent residents aged 60 years or older with complete Mini-Mental State Examination (MMSE), FRAIL, 15-item Geriatric Depression Scale (GDS-15), and Activities of Daily Living (ADL) assessments. Available records did not show that eligibility was based on a uniform research-assigned diagnosis under the Diagnostic and Statistical Manual of Mental Disorders (DSM) or the International Classification of Diseases (ICD). The source registry did not systematically record a formal cognitive diagnosis, etiological subtype, diagnosing clinician, or diagnostic date. We therefore describe the participants as institutionalized older adults and analyze cognitive function as a continuous measure rather than a diagnostic eligibility category. Because no contemporaneous screening or exclusion log was available, we do not report a screening denominator, response rate, or mutually exclusive reasons for non-inclusion.

The analytic sample included 55, 30, 29, and 68 residents in the four care cohorts. Buildings 13, 14, 21, 22, 24, and 26 contained 24, 31, 68, 29, 7, and 23 residents, respectively. Thus, building sizes were markedly imbalanced, and Building 24 contributed only seven residents.

### 2.3. Measures

The model included four constructs measured with separate instruments. Global cognitive function was assessed with the Mini-Mental State Examination (MMSE; 0–30, with higher scores indicating better cognition) [24]. For descriptive display, we prespecified four MMSE score ranges: ≥24, 18–23, 10–17, and 0–9. These ranges were used only to summarize the score distribution; they were not used to assign clinical severity, establish eligibility, or fit the continuous-score models. MMSE thresholds vary across settings and may be affected by age, education, and cultural background [25]. The source registry also contained the eight-item AD8 screening score (0–8). In a post hoc sensitivity analysis, a recorded AD8 score ≥2 was treated as the conventional screen-positive threshold [26]. Because the registry did not consistently identify whether a family informant or professional caregiver completed the AD8, this threshold indicates recorded screening status only and not a clinical diagnosis. Neither the AD8 threshold nor the MMSE display ranges were used to assign mild cognitive impairment, major neurocognitive disorder, Alzheimer’s disease, vascular dementia, or another etiological subtype. Physical frailty was assessed with the five-item FRAIL scale (Fatigue, Resistance, Ambulation, Illnesses, and Loss of weight; 0–5, with higher scores indicating greater physical frailty) [27]. The first, second, third, and fifth items rely on participant or informant history, whereas illnesses can be checked against records; psychological frailty was not assessed. Depressive symptoms were assessed with the GDS-15 (0–15, with higher scores indicating more symptoms) [28]. Scores of 0–4, 5–8, 9–11, and 12–15 were summarized as no, mild, moderate, and severe symptom levels; any depressive symptoms were defined as ≥5. The GDS-15 is a screening measure and does not diagnose major depressive disorder.

Self-care dependence was assessed with the 20-item Chinese Activities of Daily Living scale adapted by Zhang Mingyuan from the Lawton and Brody framework [29]. The physical/basic activities of daily living (PADL) subscale includes eight activities: eating, dressing, grooming, transfers, indoor mobility, toileting, continence, and bathing. The instrumental activities of daily living (IADL) subscale includes 12 activities: public transport, local mobility, food preparation, medication use, light and heavy housework, laundry, toenail care, shopping, telephone use, financial management, and staying home alone. Each item is scored from 1 (independent) to 4 (unable), producing a total of 20–80; higher scores indicate greater dependence. MMSE, GDS-15, FRAIL, and ADL assessments relied primarily on participant responses. When language-expression difficulties limited direct response, trained interviewers assisted administration and nursing staff provided collateral information when needed. All assessors completed standardized training. Sex, education, date of birth, and available clinical variables were obtained from institutional records. The participant-level assessment-time field was invalid and excluded. Age was therefore calculated from date of birth at a common reference date of 1 February 2026, near the midpoint of the verified project period; no participant-specific dates were imputed.

### 2.4. Statistical Analysis

**Figure 1:**
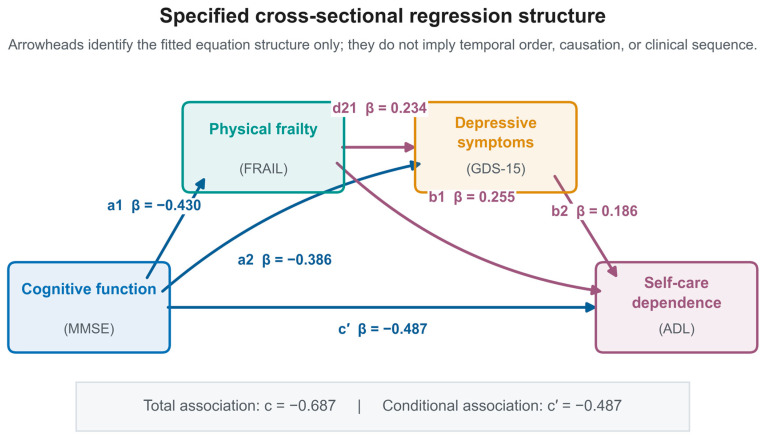
Prespecified cross-sectional regression structure with standardized coefficients. Arrowheads identify the fitted equation structure only and do not imply temporal order, causation, clinical sequence, or causal mediation. c is the standardized total association, and c′ is the standardized conditional association coefficient. No *p*-value stars are displayed.

The resident was the analytic unit. Continuous variables are summarized with means and standard deviations, and categorical variables with frequencies and percentages. Pearson correlations are reported descriptively. Internal consistency was estimated with Cronbach’s α from matched item-level records; for the MMSE, this calculation used the 12 recorded scored components. Harman’s single-factor test was treated as a limited diagnostic of shared-method variance, not proof of its absence [30]. We specified a cross-sectional regression decomposition with cognitive function as the exposure measure, physical frailty as the first ordered intervening measure, depressive symptoms as the second, and self-care dependence as the outcome (Figure 1) [31]. Ordinary least-squares regression provided the path-equation point estimates. The coefficients c and c′ denote the total and conditional cognition–self-care associations. Frailty, depression, and ordered path-product quantities are algebraic functions of the fitted regressions and do not establish a mediator process. We used 5000 participant-level resamples to calculate bias-corrected intervals (BC, not BCa) as descriptive stability summaries [32]. These intervals are not cluster-robust confidence intervals and were not used for population-level significance claims. With four care cohorts and six buildings, we summarized residential heterogeneity using intraclass correlations and six leave-one-building-out point-estimate analyses. We fitted unadjusted models and models adjusted for age, sex, and education; the adjusted model was prespecified as the primary specification.

Robustness analyses assessed variance inflation, residual normality (Shapiro–Wilk), heteroscedasticity (Breusch–Pagan), influence (Cook’s distance), Cohen’s f^2^, and the stability of point estimates under alternative model specifications. We estimated the principal competing order (depressive symptoms before frailty). To assess sensitivity to the recorded cognitive screen, we refitted the age-, sex-, and education-adjusted model among residents with AD8 scores ≥2. The comorbidity indicator, insomnia symptoms measured with the Athens Insomnia Scale (AIS), anxiety symptoms measured with the Self-Rating Anxiety Scale (SAS), loneliness measured with the UCLA Loneliness Scale (UCLA), and neuropsychiatric symptoms measured with the Neuropsychiatric Inventory (NPI) were added only as an exploratory concurrent-covariate specification, not as a causal confounder-adjustment set. Dementia subtype, medication burden, pain, nutrition, sensory impairment, detailed mobility limitation, and duration of institutionalization were unavailable. The primary ADL total was recomputed as the sum of all 20 item responses. This item sum matched the source item-workbook total for all 182 matched records. A data audit showed that a previously transferred aggregate ADL field was one point too low in 52 records; those values were corrected, and all ADL-based analyses were rerun. The PADL and IADL subscales were analyzed separately. Analyses were performed in Python 3.9, and point estimates were independently reproduced in R 4.5.3 with lavaan 0.6-21. Statistical analyses were performed using Python 3.9 and R 4.5.3 (lavaan 0.6-21). Figures were prepared using Matplotlib 3.9.4.

## 3. Results

### 3.1. Sample Characteristics

The analytic sample comprised 182 residents (Table 1). Mean age at the common 1 February 2026 reference date was 86.6 years (SD 5.8; range 70–99), and 130 participants (71.4%) were women. Twenty-four participants (13.2%) were illiterate; 49 (26.9%) had primary education; 46 (25.3%) had junior-high education; 26 (14.3%) had senior-high education; and 37 (20.3%) had college education or above. Using fixed descriptive MMSE score bands, 86 (47.3%) scored 0–9, 48 (26.4%) scored 10–17, 29 (15.9%) scored 18–23, and 19 (10.4%) scored ≥24. A recorded AD8 score ≥2 was present in 155 residents (85.2%); 163 (89.6%) had MMSE scores <24, 173 (95.1%) met at least one of these two descriptive thresholds, and 9 (4.9%) met neither. These values characterize recorded screening status and do not constitute clinical diagnoses. The source registry contained non-standardized free-text references to cognitive impairment or dementia for 28 residents; because these entries were not systematically adjudicated, they were not used to assign diagnoses or etiological subtypes. Fifty-six residents (30.8%) were frail and 76 (41.8%) pre-frail; 63 (34.6%) had GDS-15 scores ≥5. Mean ADL was 57.5 (SD 20.1; range 20–80). Cronbach’s α was 0.898 for the 12 recorded MMSE components, 0.669 for FRAIL, 0.822 for GDS-15, and 0.977 for the 20 ADL items (PADL 0.974; IADL 0.968). The first unrotated Harman factor explained 48.1% of variance, close to the conventional 50% heuristic; shared-method bias therefore cannot be excluded. Core analysis variables had no missing values. Because a contemporaneous screening/exclusion log was unavailable, selection bias among persons not represented in the analytic dataset cannot be quantified.

### 3.2. Correlations Among Study Variables

Table 2 and Figure S2 show the descriptive Pearson correlations. Lower cognitive scores co-occurred with greater self-care dependence (r = −0.687), greater frailty (r = −0.430), and more depressive symptoms (r = −0.486). Frailty co-occurred with depressive symptoms (r = 0.400) and self-care dependence (r = 0.539), whereas depressive symptoms co-occurred with self-care dependence (r = 0.525). These correlations are compatible with several cross-sectional specifications and do not establish temporal direction.

### 3.3. Prespecified Cross-Sectional Regression Specification

Table 3 summarizes the descriptive regression equations, and Figure 1 shows the standardized coefficients. In the unadjusted equations, cognitive function was inversely associated with frailty (β = −0.430). With frailty and cognition entered together, cognition (β = −0.386) and frailty (β = 0.234) were associated with depressive symptoms. In the self-care equation, cognition (β = −0.487), frailty (β = 0.255), and depressive symptoms (β = 0.186) were associated with self-care dependence. The fitted self-care equation explained 56.9% of the observed variance, and variance inflation factors ranged from 1.30 to 1.43. These coefficients describe the clustered single-institution sample.

### 3.4. Prespecified Cross-Sectional Association Decomposition

**Figure 2:**
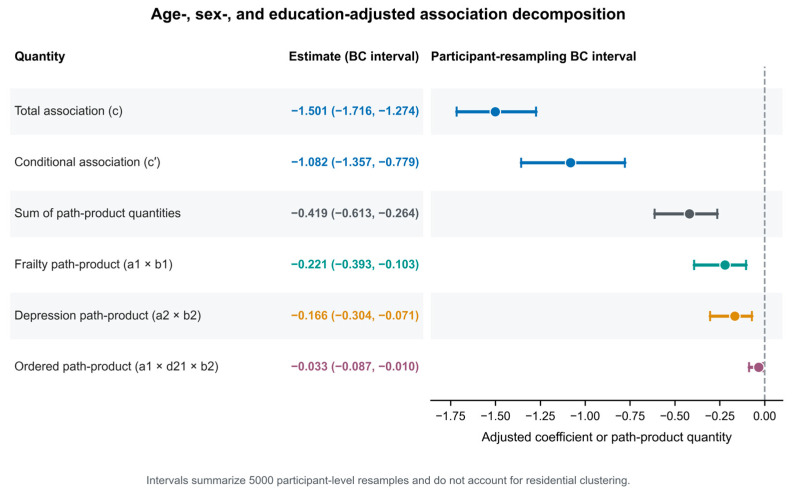
Age-, sex-, and education-adjusted cross-sectional association decomposition. Points are estimates, and horizontal bars are participant-resampling BC intervals from 5000 resamples. The intervals summarize participant-level resampling stability and do not account for residential clustering.

In the model adjusted for age, sex, and education, the total cognition–self-care association was c = −1.501 (participant-resampling BC interval −1.716 to −1.274). The conditional association coefficient was c′ = −1.082 (−1.357 to −0.779). The sum of the path-product quantities was −0.419 (−0.613 to −0.264). Under the prespecified frailty-first ordering, the frailty, depression, and ordered path-product quantities were −0.221 (−0.393 to −0.103), −0.166 (−0.304 to −0.071), and −0.033 (−0.087 to −0.010), respectively (Table 4 and Figure 2). Percentages in Table 4 are algebraic ratios to the total association and do not represent proportions mediated. The intervals summarize participant-level resampling stability and do not provide cluster-robust population inference.

The path-product quantities were smaller than the total cognition–self-care association. Their relative magnitudes changed across alternative orderings, residential omissions, and the exploratory concurrent-covariate specification. We therefore interpret the decomposition as a transparent description of a prespecified cross-sectional regression structure, not as a ranking of mechanisms or care targets.

### 3.5. Robustness and Covariate Effects

Table 4 and Figure 2 present the age-, sex-, and education-adjusted quantities. Across the prespecified MMSE display ranges, self-care dependence, frailty, and depressive symptoms were greater at lower MMSE scores (Figure S1). These ranges were used only for descriptive presentation and were not treated as clinical stages or used in the continuous-score models.

### 3.6. Sensitivity and Robustness Analyses

Table S1 summarizes the diagnostic checks. Variance inflation factors ranged from 1.30 to 1.43, and the maximum Cook’s distance was 0.10. The Shapiro–Wilk test detected residual non-normality (W = 0.985, *p* = 0.043), and the Jarque–Bera test also indicated departure from normality (*p* = 0.005). These findings motivated the descriptive resampling summaries in Table 4 but do not address residential clustering. The Breusch–Pagan test did not detect heteroscedasticity (*p* = 0.175). Cohen’s f^2^ was 0.38 for cognition, 0.12 for frailty, and 0.06 for depressive symptoms.

Residents were distributed across four care cohorts and six buildings. Self-care dependence varied substantially by cohort (mean ADL 41–70; intraclass correlation 0.42), as did cognition (0.28) and frailty (0.27). Building sizes ranged from 7 to 68 residents, and Building 24 included only seven. Each omission therefore removed a different proportion of the sample, making individual omission estimates unequally informative and potentially unstable. Because four cohorts and six buildings cannot support reliable cluster-robust population inference, Figure S3 and Table S2 report six adjusted leave-one-building-out point estimates as a descriptive sensitivity range. The frailty quantity ranged from −0.272 to −0.164, the depression quantity from −0.208 to −0.080, the ordered quantity from −0.050 to −0.018, and their sum from −0.455 to −0.370. These ranges show sensitivity to residential composition and should not be interpreted as six equally weighted replications or as cluster-derived confidence intervals.

### 3.7. Alternative-Order, Item-Level, and Expanded-Covariate Analyses

The competing depression-first ordering also produced an ordered path-product quantity (−0.055; participant-resampling BC interval −0.126 to −0.018; Table S4), confirming that the cross-sectional data do not identify a unique ordering. Among residents with recorded AD8 scores ≥2 (*n* = 155), adjusted path-product quantities retained the same direction (Table S6), although the AD8 informant source was not consistently documented. Separate PADL and IADL models produced ordered quantities of −0.014 and −0.019. In the exploratory concurrent-covariate specification, the ordered quantity was −0.013 and its percentile interval included zero (Table S5). These analyses demonstrate dependence on measurement, ordering, and model specification.

## 4. Discussion

Cognitive function, frailty, depressive symptoms, and self-care dependence were interrelated in this single-institution sample. The prespecified regression decomposition produced a large total cognition–self-care association and smaller path-product quantities. However, those quantities varied with ordering and residential composition and should not be interpreted as a causal chain.

The large conditional cognition–self-care association coefficient is compatible with the contribution of memory, attention, and executive control to everyday task performance [33]. It may also reflect unmeasured correlates such as apraxia, neuropsychiatric symptoms, and shared neurodegenerative burden [34]. Because the cohort was not defined using a uniform clinical cognitive diagnosis, this association coefficient should not be interpreted as specific to mild cognitive impairment, dementia, or any etiological subtype. The wide MMSE range, the 19 residents with scores ≥24, and the 9 residents who met neither descriptive screening threshold demonstrate heterogeneity in recorded cognitive status. The recorded-AD8 ≥2 sensitivity analysis did not materially change the direction of the reported quantities, but it cannot validate a screening threshold, identify a diagnostic group, or resolve measurement or selection bias.

The frailty path product was the largest of the three path-product quantities in the primary specification. Physical frailty, cognitive function, and ADL dependence share biological and functional correlates [35,36], while FRAIL and ADL also overlap in mobility-related content. Their correlation (r = 0.54) indicates substantial but incomplete overlap. Separate PADL and IADL analyses did not remove this concern, and the building-omission range showed that residential composition influenced the frailty quantity.

The depression path product remained distinct from the frailty quantity in the primary specification. A depression-first ordering also produced an ordered path product, however, so the observed data cannot establish which condition precedes the other. GDS-15 scores represent symptom burden rather than depressive disorder, and very low MMSE scores may reduce response reliability.

Our findings complement studies that modeled frailty, depressive symptoms, cognition, and daily functioning in different orders and settings. The present analysis provides hypothesis-generating evidence about competing cross-sectional association structures, not a test of a temporal pathway.

Strengths include complete data for the core variables, item-level ADL reconciliation, transparent sensitivity analyses, and independent reproduction of point estimates in Python and R. The Supplementary Materials provide the analysis code, variable dictionary, and exact model specifications. Assessors received standardized training, and participant responses were supplemented with interviewer assistance or nursing-staff collateral information when language-expression difficulties limited direct response. The first Harman factor explained 48.1% of variance, close to the conventional heuristic; shared-method variance therefore cannot be excluded.

Several limitations require emphasis. First, cross-sectional data cannot establish temporal or causal mediation, and the competing-order result demonstrates non-uniqueness. Second, the single-institution sample, four cohorts, six buildings, and marked cluster-size imbalance (including Building 24, *n* = 7) preclude reliable cluster-robust population inference. Third, the source registry did not systematically contain a research-assigned DSM- or ICD-based cognitive diagnosis, etiological subtype, diagnosing clinician, or diagnostic date. We therefore cannot distinguish mild cognitive impairment, major neurocognitive disorder, Alzheimer’s disease, vascular dementia, mixed dementia, or other cognitive disorders. Fourth, the MMSE display ranges were not education-adjusted, and the AD8 informant source was not consistently documented. Fifth, no contemporaneous screening or exclusion log or mode-specific counts for interviewer assistance and collateral information were retained. Sixth, assessors received standardized training, but no inter-rater calibration statistic was available. Seventh, GDS-15 and several FRAIL items depend on report quality, creating a risk of differential measurement error. Eighth, FRAIL and ADL share physical-function content. Finally, medication use, pain, nutrition, sensory impairment, detailed mobility, and duration of institutionalization were unavailable. The exploratory concurrent-covariate model was not a causal confounder analysis and further illustrates model dependence.

Frailty and depressive-symptom screening may provide complementary descriptive information about residents with cognitive and self-care difficulties. These associations do not show that treating either condition will preserve self-care or that one should be addressed before the other.

### Future Research Directions

Prospective multicenter studies should repeatedly measure cognition, frailty, depressive symptoms, and PADL/IADL so that competing temporal orders can be evaluated with longitudinal models. Samples should include enough institutions and clusters for multilevel estimation, document cognitive diagnoses and severity when clinically established, and measure medication use, pain, nutrition, mobility, sensory impairment, and length of stay. Studies involving residents with very low cognitive scores should prespecify validated self-report, interviewer-assisted, and proxy procedures and evaluate measurement invariance across response modes. Prospectively registered randomized trials may subsequently determine whether frailty or depression interventions improve self-care and whether treatment order matters.

## 5. Conclusions

Among residents of this long-term care institution, lower cognitive scores co-occurred with greater self-care dependence, frailty, and depressive symptoms. The prespecified decomposition produced smaller path-product quantities under one assumed ordering, but competing orderings and building-omission analyses showed that the data cannot identify temporal sequence, causal mediation, or an intervention target. Larger longitudinal, multicenter studies with validated response procedures are needed.

## Figures and Tables

**Table 1:** Characteristics of the study sample (*n* = 182).

Variable	Value	Range
Age (years), mean ± SD	86.6 ± 5.8	70–99
**Sex, *n* (%)**		
Female	130 (71.4)	
Male	52 (28.6)	
**Education, *n* (%)**		
Illiterate	24 (13.2)	
Primary	49 (26.9)	
Junior high	46 (25.3)	
Senior high	26 (14.3)	
College or above	37 (20.3)	
Cognitive function (MMSE), mean ± SD	10.5 ± 9.4	0–30
**Fixed descriptive MMSE score band, *n* (%)**		
≥24	19 (10.4)	
18–23	29 (15.9)	
10–17	48 (26.4)	
0–9	86 (47.3)	
Recorded AD8 score, mean ± SD	4.7 ± 2.5	0–8
Recorded AD8 score ≥2, *n* (%)	155 (85.2)	
Frailty (FRAIL), mean ± SD	1.7 ± 1.4	0–5
**FRAIL category, *n* (%)**	Robust: 50 (27.5)	
	Pre-frail: 76 (41.8)	
	Frail: 56 (30.8)	
Depressive symptoms (GDS-15), mean ± SD	3.1 ± 3.1	0–13
Any depressive symptoms, *n* (%)	63 (34.6)	
Self-care dependence (ADL; higher = more dependent), mean ± SD	57.5 ± 20.1	20–80

AD8, eight-item informant interview for cognitive screening; MMSE, Mini-Mental State Examination; FRAIL, frailty scale; GDS-15, 15-item Geriatric Depression Scale; ADL, activities of daily living; SD, standard deviation. Percentages may not sum to 100.0 because of rounding. Screening thresholds are descriptive and do not establish a clinical diagnosis.

**Table 2:** Descriptive Pearson correlations among the core study variables and covariates (*n* = 182).

Variable	1	2	3	4	5	6
1. Cognitive function	1					
2. Frailty	−0.430	1				
3. Depressive symptoms	−0.486	0.4	1			
4. Self-care dependence	−0.687	0.539	0.525	1		
5. Age	−0.065	0.191	0.177	0.217	1	
6. Education (years)	0.192	−0.036	0.053	−0.077	−0.061	1

Correlation coefficients are reported descriptively for the analytic sample; no cluster-robust hypothesis tests are presented. Cognitive function was measured with the MMSE, frailty with the FRAIL scale, depressive symptoms with the GDS-15, and self-care dependence with the ADL scale (higher scores indicate greater dependence).

**Table 3:** Descriptive regression coefficients for the unadjusted cross-sectional specification (*n* = 182).

Outcome	Predictor	B	β
Frailty (R^2^ = 0.185)	Cognitive function	−0.062	−0.430
Depressive symptoms (R^2^ = 0.281)	Cognitive function	−0.126	−0.386
	Frailty	0.528	0.234
Self-care dependence (R^2^ = 0.569)	Cognitive function	−1.045	−0.487
	Frailty	3.785	0.255
	Depressive symptoms	1.221	0.186

B, unstandardized coefficient; β, standardized coefficient. These unadjusted coefficients describe the observed single-institution analytic sample and are not cluster-robust population inference. Cognitive function was measured with the MMSE, frailty with the FRAIL scale, depressive symptoms with the GDS-15, and self-care dependence with the ADL scale. Equations were fitted in sequence to define the displayed regression specification.

**Table 4:** Covariate-adjusted cross-sectional association decomposition between cognitive function and self-care dependence (5000 participant-level resamples).

Quantity	Estimate	BC Interval *	% of Total †
Total association (c)	−1.501	−1.716, −1.274	100
Conditional association (c′)	−1.082	−1.357, −0.779	72.1
Sum of path-product quantities	−0.419	−0.613, −0.264	27.9
Frailty path product (a1 × b1)	−0.221	−0.393, −0.103	14.7
Depression path product (a2 × b2)	−0.166	−0.304, −0.071	11
Ordered path product (a1 × d21 × b2)	−0.033	−0.087, −0.010	2.2

c, total association; c′, conditional association coefficient. * Participant-resampling BC intervals describe resampling stability and are not cluster-robust confidence intervals. † Percentages are algebraic ratios to the total association and are not proportions mediated. Cognition denotes cognitive function (MMSE), frailty the FRAIL scale, depression depressive symptoms (GDS-15), and self-care self-care dependence (ADL; higher scores indicate greater dependence).

## Data Availability

The complete analytic dataset is not publicly available because of privacy and ethical restrictions; requests may be directed to the corresponding author and are subject to institutional and ethics approval.

## Supplementary Materials

The following supporting information can be downloaded at: https://www.mdpi.com/article/10.3390/healthcare14152428/s1, The supplementary file contains Figures S1–S3 and Tables S1–S6, including descriptive distributions, a correlation matrix, residential-structure and building-omission sensitivity analyses, diagnostic checks, and additional sensitivity analyses.
