# Supplementary material for: Cross-Sectional Associations of Cognitive Function, Frailty, Depressive Symptoms, and Self-Care Dependence in Residents of a Long-Term Care Institution
Source: Healthcare (Basel). 2026 Aug 6;14(15):2428. doi: 10.3390/healthcare14152428 (PMC13465258; doi:10.3390/healthcare14152428)
Supplement: Supplementary file 1 [file healthcare-14-02428-s001.zip › healthcare-4400424-supplementary.pdf]

Supplementary Materials

Cross-Sectional Associations of Cognitive Function, Frailty, Depressive Symptoms, and Self-Care Dependence in Residents of a Long-Term Care Institution

Supplementary Figures

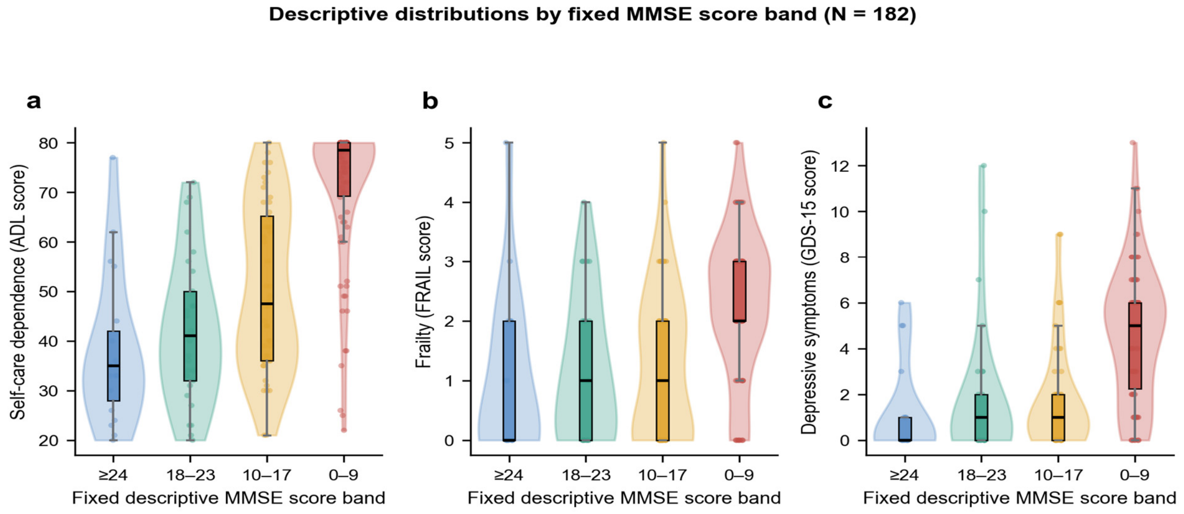

Figure S1. Descriptive distributions of self-care dependence measured with the Activities of Daily Living scale (ADL), frailty measured with the FRAIL scale, and depressive symptoms measured with the 15-item Geriatric Depression Scale (GDS-15) across four fixed descriptive Mini-Mental State Examination (MMSE) score bands (n = 182). Boxes show the median and interquartile range; whiskers extend to the most extreme values within 1.5 × the interquartile range, and all individual observations are shown over the violin density. These bands are descriptive and are not clinical disease stages.

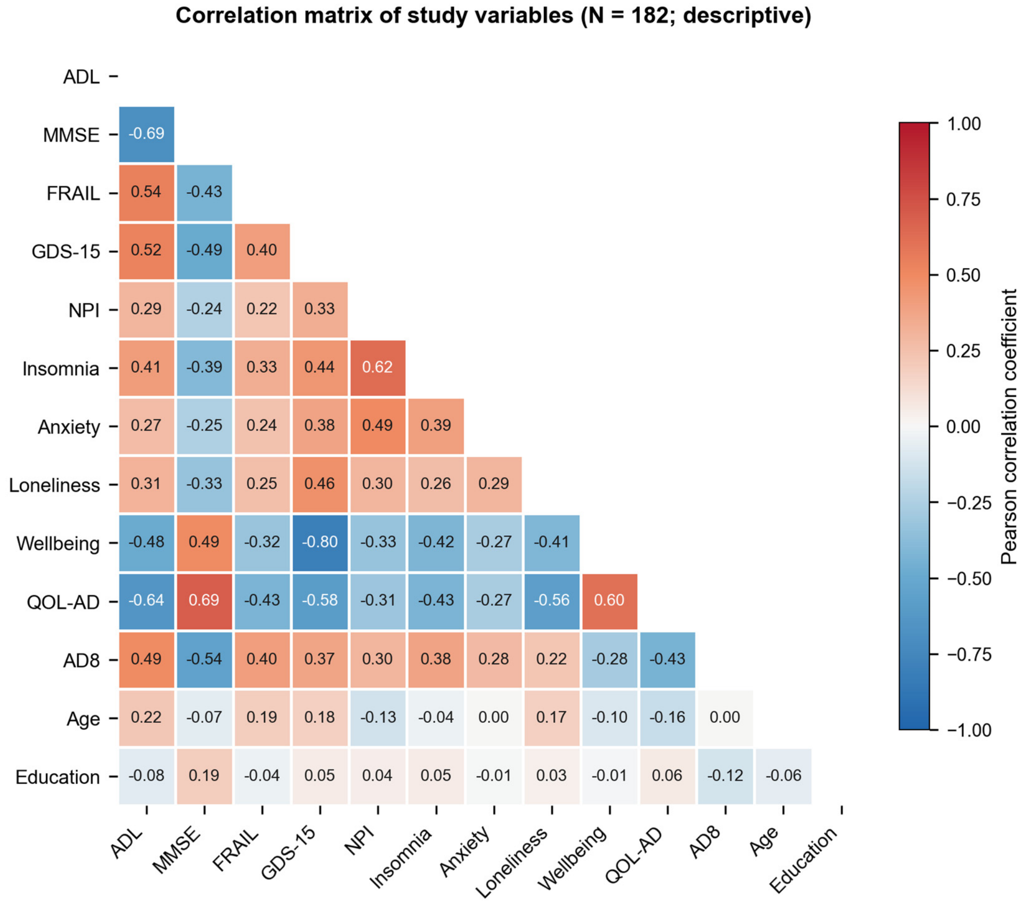

Figure S2. Descriptive Pearson correlation matrix of all assessed variables (n = 182). Cell shading and labels encode the correlation coefficient; no hypothesis-test annotations are displayed.

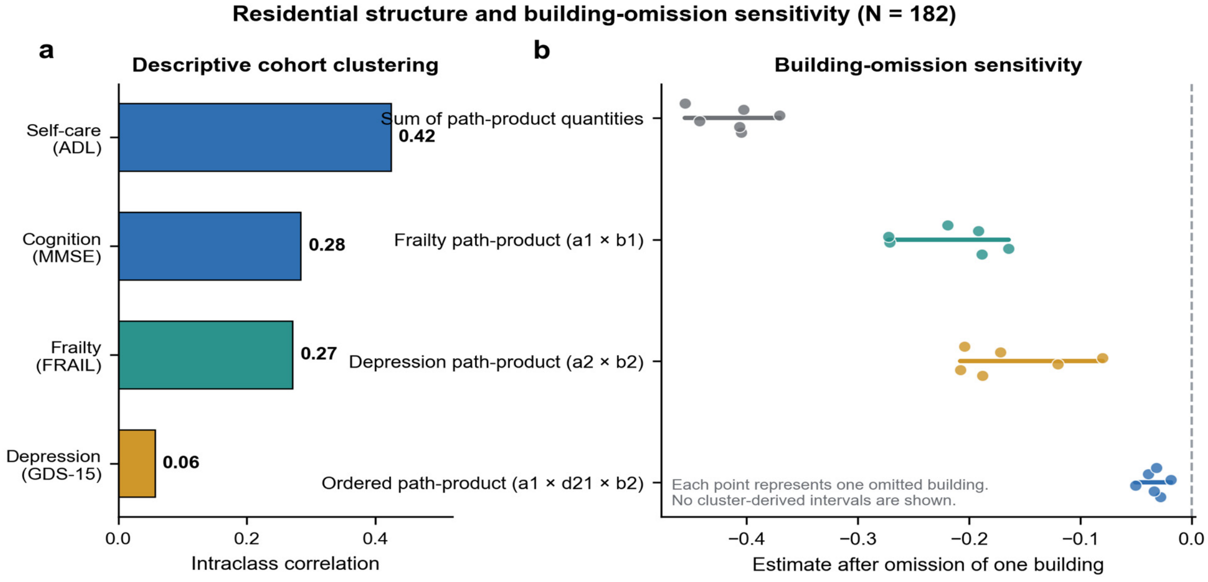

Figure S3. Residential structure and building-omission sensitivity (n = 182). (a) Intraclass correlations describe cohort-level variation in self-care dependence (ADL), cognitive function (MMSE), frailty (FRAIL), and depressive symptoms (GDS-15). (b) Each point is an adjusted point estimate after omitting one of six buildings. Ranges describe residential-composition sensitivity; no cluster-derived confidence intervals or p values are shown.

Supplementary Tables

Table S1. Diagnostic checks and descriptive fit quantities for the self-care (ADL) regression equation (ADL regressed on MMSE, FRAIL, and GDS-15; n = 182).

Check	Statistic	Interpretation
Residual normality (Shapiro-Wilk)	W = 0.985, p = 0.043	Residual-normality diagnostic suggests departure; participant-resampling summaries are descriptive
Residual normality (Jarque-Bera)	10.78, p = 0.005	Residual-normality diagnostic suggests departure; participant-resampling summaries are descriptive
Homoscedasticity (Breusch-Pagan)	LM = 4.96, p = 0.175	No evidence of heteroscedasticity detected
Influence (maximum Cook's distance)	0.10	No observation exceeds 1
Multicollinearity (maximum VIF)	1.43	Well below 5
Effect size, cognition (Cohen's f^2)	0.38	Large
Effect size, frailty (Cohen's f^2)	0.12	Small to medium
Effect size, depression (Cohen's f^2)	0.06	Small
Model fit (R2 / adjusted R2)	0.569 / 0.562	Outcome model

VIF, variance inflation factor; Cohen's f^2 benchmarks: 0.02 small, 0.15 medium, 0.35 large. These checks describe the fitted individual-level equation and do not establish cluster-robust population inference.

Table S2. Building-omission sensitivity of covariate-adjusted path-product quantities (n = 182).

Quantity	Full analytic sample	Range after omitting one building	Interpretation
Sum of path-product quantities	-0.419	-0.455 to -0.370	Residential-composition sensitivity
Frailty path-product (a1 × b1)	-0.221	-0.272 to -0.164	Residential-composition sensitivity
Depression path-product (a2 × b2)	-0.166	-0.208 to -0.080	Residential-composition sensitivity
Ordered path-product (a1 × d21 × b2)	-0.033	-0.050 to -0.018	Residential-composition sensitivity

Values are point estimates. The range is calculated from six analyses, each omitting one building. It is a descriptive residential-composition sensitivity analysis, not a confidence interval or hypothesis test.

Table S3. Item-level reconciliation and internal consistency (n = 182 matched records).

Scale	Items/components	Cronbach's α	Exact total match	Total differed
MMSE	12	0.898	182	0

FRAIL	5	0.669	182	0
GDS-15	15	0.822	182	0
ADL total	20	0.977	182	0
PADL	8	0.974	—	—
IADL	12	0.968	—	—

Note: The item-level workbook contained 183 records; analyses were restricted to the 182 records matched to the analytic cohort, leaving one unmatched item-only record outside the analysis. MMSE, FRAIL, and correctly reverse-coded GDS-15 item sums matched the analysis totals in all 182 matched records. The 20-item ADL sum matched the source item-workbook total for all 182 records. A previously transferred aggregate ADL field was one point too low in 52 records and equal in 130; the 52 values were corrected, and all revised ADL analyses use the verified item sum.

Table S4. Alternative-order and corrected ADL/PADL/IADL association-decomposition sensitivity analyses (age, sex, and education adjusted; 5000 participant-level resamples). Participant-resampling BC intervals are descriptive resampling summaries, not cluster-robust confidence intervals. The sum of path-product quantities is invariant to ordering and is reported once.

Specification/outcome	Quantity	Estimate	Participant-resampling BC interval
Corrected total ADL; frailty-first specification	Sum of path-product quantities	-0.419	-0.610, -0.264
Corrected total ADL; frailty-first specification	Ordered path-product quantity	-0.033	-0.082, -0.009
Corrected total ADL; depression-first specification	Ordered path-product quantity	-0.055	-0.126, -0.018
PADL; frailty-first specification	Sum of path-product quantities	-0.177	-0.269, -0.104
PADL; frailty-first specification	Ordered path-product quantity	-0.014	-0.038, -0.004
IADL; frailty-first specification	Sum of path-product quantities	-0.243	-0.364, -0.146
IADL; frailty-first specification	Ordered path-product quantity	-0.019	-0.051, -0.005

Table S5. Exploratory concurrent-covariate specification (age, sex, education, comorbidity indicator, Athens Insomnia Scale [AIS], Self-Rating Anxiety Scale [SAS], UCLA Loneliness Scale [UCLA], and Neuropsychiatric Inventory [NPI]; participant-resampling BC and percentile intervals). These intervals describe resampling stability and are not cluster-robust confidence intervals. This is not a causal confounder-adjustment model.

Quantity	Estimate	Participant-resampling BC interval	Participant-resampling percentile interval
Frailty path-product	-0.156	-0.323, -0.057	-0.311, -0.052
Depression path-product	-0.092	-0.205, -0.027	-0.191, -0.020
Ordered path-product quantity	-0.013	-0.045, -0.001	-0.037, 0.002
Sum of path-product quantities	-0.260	-0.437, -0.135	-0.433, -0.132

Table S6. Recorded cognitive-screening-status sensitivity analysis (age-, sex-, and education-adjusted cross-sectional association decomposition; participant-resampling BC intervals presented as descriptive resampling summaries, not cluster-robust confidence intervals).

Quantity	Complete cohort (n = 182)	Recorded AD8 ≥2 (n = 155)
Frailty path-product	-0.221 (-0.400, -0.103)	-0.198 (-0.389, -0.082)
Depression path-product	-0.166 (-0.302, -0.069)	-0.156 (-0.318, -0.060)
Ordered path-product quantity	-0.033 (-0.082, -0.009)	-0.033 (-0.094, -0.008)
Sum of path-product quantities	-0.419 (-0.610, -0.264)	-0.387 (-0.599, -0.227)
Conditional association	-1.082 (-1.347, -0.790)	-1.170 (-1.446, -0.843)
Total association	-1.501 (-1.712, -1.272)	-1.556 (-1.790, -1.312)

Values are point estimates with participant-resampling BC intervals from 5000 resamples. These intervals describe resampling stability and are not cluster-robust confidence intervals. AD8 ≥2 is used only as a recorded screening threshold for this post hoc sensitivity analysis and does not establish a Diagnostic and Statistical Manual of Mental Disorders (DSM) or International Classification of Diseases (ICD) diagnosis, or an etiological subtype. The source registry did not consistently record the AD8 informant type.

Abbreviations: AD8, eight-item informant interview for cognitive screening; ADL, activities of daily living; AIS, Athens Insomnia Scale; GDS-15, 15-item Geriatric Depression Scale; IADL, instrumental activities of daily living; MMSE, Mini-Mental State Examination; NPI, Neuropsychiatric Inventory; PADL, physical/basic activities of daily living; SAS, Self-Rating Anxiety Scale; UCLA, UCLA Loneliness Scale.
